# A hand hygiene intervention to decrease infections among children attending day care centers: design of a cluster randomized controlled trial

**DOI:** 10.1186/1471-2334-13-259

**Published:** 2013-06-03

**Authors:** Tizza P Zomer, Vicki Erasmus, Nico Vlaar, Ed F van Beeck, Aimée Tjon-A-Tsien, Jan Hendrik Richardus, Hélène ACM Voeten

**Affiliations:** 1 Department of Infectious Disease Control, Municipal Public Health Service Rotterdam-Rijnmond, Rotterdam, The Netherlands; 2 Department of Public Health, Erasmus MC, University Medical Center Rotterdam, Rotterdam, The Netherlands; 3 Department of Environment and Hygiene, Municipal Public Health Service Rotterdam-Rijnmond, Rotterdam, The Netherlands

## Abstract

**Background:**

Day care center attendance has been recognized as a risk factor for acquiring gastrointestinal and respiratory infections, which can be prevented with adequate hand hygiene (HH). Based on previous studies on environmental and sociocognitive determinants of caregivers’ compliance with HH guidelines in day care centers (DCCs), an intervention has been developed aiming to improve caregivers’ and children’s HH compliance and decrease infections among children attending DCCs. The aim of this paper is to describe the design of a cluster randomized controlled trial to evaluate the effectiveness of this intervention.

**Methods/design:**

The intervention will be evaluated in a two-arm cluster randomized controlled trial among 71 DCCs in the Netherlands. In total, 36 DCCs will receive the intervention consisting of four components: 1) HH products (dispensers and refills for paper towels, soap, alcohol-based hand sanitizer, and hand cream); 2) training to educate about the Dutch national HH guidelines; 3) two team training sessions aimed at goal setting and formulating specific HH improvement activities; and 4) reminders and cues to action (posters/stickers). Intervention DCCs will be compared to 35 control DCCs continuing usual practice. The primary outcome measure will be observed HH compliance of caregivers and children, measured at baseline and one, three, and six months after start of the intervention. The secondary outcome measure will be the incidence of gastrointestinal and respiratory infections in 600 children attending DCCs, monitored over six months by parents using a calendar to mark the days their child has diarrhea and/or a cold. Multilevel logistic regression will be performed to assess the effect of the intervention on HH compliance. Multilevel poisson regression will be performed to assess the incidence of gastrointestinal and respiratory infections in children attending DCCs.

**Discussion:**

This is one of the first DCC intervention studies to assess HH compliance of both caregivers and children, as well as the incidence of gastrointestinal and respiratory infections in children, as outcome measures. When an effect of the intervention on improving HH compliance and/or reducing incidence of infections is shown, (inter)national dissemination of the intervention in other DCCs may be considered.

**Trial registration:**

Netherlands trial registry: NTR3000

## Background

Attendance at child day care centers (DCCs) has been recognized as a risk factor for acquiring gastrointestinal and respiratory infections [1-3]. These infections can cause distress for both the children and their parents, incur costs for health care and parental work absence, and result in secondary transmission [4-6]. Hand hygiene (HH) is a simple and effective measure to prevent infections [7,8]. In the Dutch national HH guidelines for DCCs, the activities for which HH is indicated are outlined [9]. However, compliance with HH guidelines is generally low; hands are adequately washed in Dutch DCCs in less than half of all HH opportunities (compliance 42%) [10]. Several HH interventions have been developed to decrease infections in DCCs [11-17]. However, these interventions show varying effects [18] and are not developed according to a stepwise behavioral approach taking into account the underlying determinants of HH behavior [19]. Interventions developed based on these determinants are more likely to be effective in the long term [20]. A study on HH in hospitals has shown that interventions have more effect when a combination of multiple determinants is addressed [21].

We assessed environmental and sociocognitive determinants of caregivers’ compliance with HH guidelines [10,22], and have used the results of our studies to develop a multi-component intervention aiming to improve caregivers’ and children’s HH compliance and decrease infections among children attending DCCs. The intervention consists of the following four components: 1) products necessary for HH (i.e. dispensers and refills for paper towels, soap, alcohol-based hand sanitizer, and hand cream); 2) training to educate about the Dutch national HH guidelines; 3) two team training sessions aimed at goal setting and formulating specific HH improvement activities; and 4) reminders and cues to action (i.e. posters and stickers). The four components of the intervention together could potentially result in better HH compliance and fewer gastrointestinal and respiratory infections among children attending DCCs. However, before (inter)national dissemination of the intervention in other DCCs can be considered, it is necessary to evaluate the effectiveness of the intervention. The objective of this paper is to describe the design of a cluster randomized controlled trial to evaluate the effectiveness of the HH intervention.

## Methods/design

### Objectives and hypotheses

The study objective is to evaluate the effectiveness of a HH intervention in DCCs. Our hypotheses are that HH compliance of caregivers and children in intervention DCCs will be significantly higher than in control DCCs, and that children attending intervention DCCs will have significantly less gastrointestinal and respiratory infections than children attending control DCCs.

### Study design

The intervention will be tested in a two-arm cluster randomized controlled trial, to be conducted among 71 child DCCs; 36 intervention DCCs receive the intervention, while 35 control DCCs continue usual practice.

### Setting

The study will be conducted among DCCs in the Netherlands in the regions of Rotterdam-Rijnmond, Leiden and Gouda. This is a mixed urban–rural area with about 1.5 million inhabitants (of the total of approximately 17 million inhabitants in the Netherlands). In this area around 25,000 children attend about 390 DCCs (unpublished data 2008).

### Intervention development

Our previous studies concerning environmental and sociocognitive determinants of caregivers’ compliance with HH guidelines in DCCs [10,22] were used to develop a multi-component HH intervention for DCCs. The intervention targets caregivers’ sociocognitive determinants such as guideline knowledge and awareness, perceived importance of performing HH, caregivers’ own perceived ability to perform HH when needed (i.e. perceived behavioral control), and habit (Table 1). In addition, with the provision of HH products the intervention targets environmental determinants (Table 1).

**Table 1:** Intervention components and targeted determinants of hand hygiene (HH) behavior

**Intervention component**	**Targeted determinants of HH behavior**
1. Provision of HH products: dispensers and refills for paper towels, soap, alcohol-based hand sanitizer and hand cream	Environmental determinants, especially the availability of paper towels
2. Training to educate about the Dutch national HH guidelines; information booklet	Guideline knowledge and awareness, perceived HH importance
3. Two team training sessions aimed at goal setting and formulating specific HH improvement activities	Perceived HH importance, perceived behavioral control
4. Posters and stickers as reminders and cues to action	Guideline knowledge and awareness, habit

Our study on the environmental determinants of caregivers’ HH compliance showed that hands are most frequently washed when only paper towels are available compared to only fabric towels or a combination of both paper and fabric towels [10]. Therefore, the intervention includes the provision of paper towel dispensers and refills. We also provide dispensers and refills for liquid soap, alcohol-based hand sanitizer, and hand cream to ensure that all necessary products for HH are available.

Our study on sociocognitive determinants demonstrated that the following determinants were related to HH compliance of caregivers: knowledge and awareness of the guidelines, perceived importance of performing HH, perceived behavioral control (i.e. caregivers’ own perceived ability to perform HH when needed), and habit [22]. To improve knowledge and awareness of the HH guidelines and to increase perceived importance of HH, a one hour training session which incorporates the following topics was developed: transmission of infectious diseases, importance of HH at DCCs, the different activities outlined in the guidelines for which HH is indicated both for caregivers and children, and the techniques for performing HH using soap and water or alcohol-based hand sanitizer. The training session also includes an exercise using UV Glow Cream (Deb Benelux, Inc.) and a UV lamp to demonstrate the difference between quick and thorough hand washing. After the training session all participants, as well as caregivers who cannot attend, receive a booklet that outlines the content of the training about the HH guidelines.

To increase perceived behavioral control, two team training sessions were developed. The aim of these training sessions is to get team members to formulate team goals concerning HH and specific activities to improve HH of caregivers and children at their DCC. During the first training session, team members discuss current HH compliance, goal setting for future compliance, barriers and facilitators, and strengths of their team. During the second training session, which takes place about one month later, the interlaying period is reflected on and the following topics are discussed: rating of HH compliance of the team, improvements made so far, remaining difficulties and what is needed to address these, communication in case HH is not performed, and how to maintain the achieved results in the long term. The team training sessions are guided by trained coaches and are based on similar HH training sessions developed for Dutch hospitals [23].

The intervention also includes reminders and cues to action to stimulate HH to become habitual behavior. For both caregivers and children a poster with the activities for which HH is indicated and a poster with the technique for adequate hand washing was developed, as well as reminder stickers. The posters and stickers were developed in collaboration with the department of Industrial Design of the Delft University of Technology in the Netherlands.

The intervention DCCs will be compared to control DCCs continuing usual practice. After data collection, the control DCCs will also be offered the intervention to motivate participation in the study.

### Participants

Of 390 DCCs, 122 participated in our previous study on environmental and sociocognitive determinants of caregivers’ compliance with HH guidelines [10,22]. Of these 122 DCCs, 71 will participate in the trial to evaluate the effectiveness of the intervention. In Dutch DCCs, children aged three months to four years are cared for in groups or classes and each group of children has its own room where the daily activities take place (i.e. classrooms). In each participating DCC (both intervention and control), data will be collected in two of these groups, even if the DCC has more than two groups in total. Study participants will be caregivers (excluding interns) working in these two groups and children attending these groups. Inclusion criteria for the children are: aged at start of the trial between six months and 3.5 years; attending the DCC at least two days a week; intending to attend the DCC throughout the study period; and consenting Dutch speaking parents with access to email or regular post. Exclusion criteria for the children are: chronic illness or medication that would predispose them to infection; a sibling taking part in the trial (i.e. one child per family); and starting to attend the DCC after the start of the trial.

### Randomization

Stratified randomization is performed by assigning each DCC to one of six strata based on size (i.e. small < 46 children per day versus large ≥ 46 children per day) and geographic location (i.e. highly urban versus urban versus slightly/non-urban). DCCs are assigned to either intervention or control group by means of computer generation with a 1:1 ratio in each of the strata.

### Outcome measures

#### Primary outcome measure: observed HH compliance

The primary outcome measure is observed compliance of caregivers with HH guidelines. Compliance is defined as the number of HH actions divided by the total number of opportunities for which HH is indicated according to the Dutch national guidelines. According to these guidelines, HH is mandatory for caregivers before touching/preparing food, before caregivers themselves eat or assist children with eating, and before wound care; and after diapering, after toilet use/wiping buttocks, after caregivers themselves cough/sneeze/wipe their own nose, after contact with body fluids (e.g. saliva, vomit, urine, blood, or mucus when wiping children’s noses), after wound care, and after visibly soiled hands [9]. For these HH indications it will be observed whether or not HH is performed. As observations cannot take place in the caregivers’ lavatory, HH after toilet use will only be observed after assisting a child with toilet use and not after toilet use by caregivers themselves. HH is defined as washing hands with water and soap followed by hand drying, or use of an alcohol-based hand sanitizer. An alcohol-based hand sanitizer cannot be used when hands are visibly soiled; in this case, hand washing with soap and water is required.

Although the primary outcome measure is HH compliance of caregivers, it will also be observed whether caregivers supervise children to wash their hands, because the HH indications outlined in the guidelines also apply to children [9]. It will be observed whether caregivers supervise children to wash their hands before eating/preparing food, after toilet use, after playing outside, and after visibly soiled hands. Children should wash their hands with water and soap followed by hand drying. For babies and toddlers who cannot wash their hands themselves yet, caregivers can perform HH by using a wet cloth with soap on one side and only water on the other side [9].

Compliance will be assessed with direct unobtrusive observation by trained observers before start of the intervention (T0) and one (T1), three (T2), and six (T3) months after start of the intervention. At each measurement time point (i.e. T0, T1, T2 and T3) the aim is to observe, during a single day in each DCC, three caregivers in the two groups participating in the study and to observe each caregiver for two hours. One observer will observe one caregiver at a time, as well as the children of which HH is supervised by that caregiver. Observations will take place during routine care activities in common rooms including the diaper-changing room, the kitchen and the indoor/outdoor playgrounds. The observers will collect data using personal digital assistants (PDAs) for electronic on-site data entry. Data will be collected using the World Health Organization HH observation method [24], adapted for use in child DCCs.

#### Secondary outcome measure: incidence of gastrointestinal and respiratory infections in children

The secondary outcome measure is the incidence of gastrointestinal and respiratory infections in children attending DCCs. The aim is that 600 parents will monitor disease incidence in their child using strict definitions for diarrhea and a cold. Diarrhea is defined as at least two watery or unusually loose bowel motions in 24 hours [15]. A cold is defined as a blocked or runny nose with at least one of the following symptoms: coughing, sneezing, fever, sore throat, or earache.

Disease incidence will be assessed by parents using a paper calendar to mark the days their child has diarrhea and/or a cold. Each calendar page includes the definitions of illness. The paper calendar will facilitate record keeping and minimize recall bias. Parents will be contacted every two weeks by email and by regular post to enter the calendar page in an online version of the calendar or to send it in by regular post using a free-of-charge return envelop. The email will contain a link to the online calendar and parents will receive a password to ensure confidentiality. Parents who do not respond will receive a reminder email after one week, after two weeks they will receive a reminder letter, and after three weeks, if by then they still have not replied, they will be contacted by telephone. Monitoring of disease incidence by parents will last six months during which the intervention will be implemented in phases and baseline and follow-up data will be collected. In total, parents will be asked to return 14 calendar pages, with each page covering two weeks. To stimulate response parents will receive small incentives during the six months data collection (e.g. inflatable beach ball) and parents who return all calendar pages will receive a larger incentive at the end of the trial (i.e. tickets for the whole family for an amusement park for children).

### Intervention implementation and data collection

The intervention will be implemented in the 36 intervention DCCs in phases over a period of six months. Timing of the intervention will be during the winter months, namely from mid-September until the end of March, when most gastrointestinal and respiratory infections occur. In all participating DCCs (both intervention and control), data will be collected in two groups. In each intervention DCC, due to budget restrictions, only the two groups where data are collected will receive the HH products and refills for six months. To facilitate support from the management and to stimulate cultural changes concerning HH, the training sessions and posters/stickers will be offered to the whole intervention DCC.

**Figure 1:**
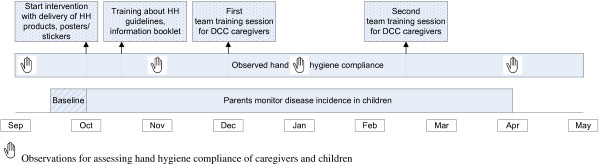
Timeline of data collection and phased implementation of a hand hygiene intervention at child day care centers (DCCs).

Figure 1 shows the timeline of phased implementation of the intervention and data collection. Baseline compliance (T0) and baseline incidence rates will be collected prior to start of the intervention. The intervention will start with the delivery of the dispensers with refills and posters/stickers. Shortly after that the training to educate about the national HH guidelines will be given. Compliance will be observed again one month after start of the intervention (T1) and this will be followed by the first team training session. Compliance will then be observed once more three months after start of the intervention (T2) after which the second team training session will be given. The final compliance observations will take place after the second team training session and six months after start of the intervention (T3).

### Blinding

DCCs will not be blinded to treatment arms; the managers will be informed whether their DCC is allocated to the intervention or control group. Although the observers who collect compliance data and parents who monitor disease incidence will not be informed whether the DCC is in the intervention or control group, they will probably recognize the intervention materials.

### Data analyses

First, descriptive analyses will be performed to assess the effect of the intervention on observed HH compliance and on incidence of gastrointestinal and respiratory infections in children. Compliance with HH guidelines will be calculated by dividing the number of HH actions by the total number of opportunities for which HH is indicated according to the Dutch national guidelines. Incidence of gastrointestinal and respiratory infections will be calculated by dividing the number of illness episodes by the total number of days at risk.

To account for clustering of the data within caregivers/children and within DCCs, multilevel analyses will be performed. The effect of the intervention on observed HH compliance will be analyzed using multilevel logistic regression. Multilevel poisson regression will be used to analyze the effect of the intervention on incidence of gastrointestinal and respiratory infections in children. If necessary, baseline differences between intervention and control DCCs will be corrected for, as well as for possible confounders at the level of the DCCs, caregivers or children.

### Sample size calculation

#### Primary outcome measure: observed HH compliance

HH compliance is expected to increase due to the intervention from 30% at baseline to 60% six months after start of the intervention. To detect this increase we would need a sample size of 20 DCCs in a two-arm cluster randomized controlled trial (10 intervention and 10 control DCCs). This is based on 80% power with a two-sided alpha of 0.05, assuming 60 observed HH opportunities per DCC per measurement time point (i.e. T0, T1, T2 and T3) and taking into account clustering of data within DCCs.

#### Secondary outcome measure: incidence of gastrointestinal and respiratory infections in children

To be able to detect 25% reduction in incidence of gastrointestinal infections of three per year and 15% reduction in incidence of respiratory infections of nine per year, we would need a sample size of 60 DCCs (30 intervention and 30 control DCCs) and disease monitoring of 600 children (10 children per DCC) for six months. This is based on 80% power with a two-sided alpha of 0.05, assuming 10 children per DCC and taking into account clustering of data within children and within DCCs. The assumed reduction in disease incidence seems to be realistic, given the pooled estimates of 39% and 31% reduction in gastrointestinal illness [7,25], and a pooled estimate of 21% reduction in respiratory illness [25].

To be able to detect an effect of the intervention on both our primary and secondary outcome measure, it is necessary to include at least 60 DCCs in the cluster randomized controlled trial (30 intervention and 30 control DCCs) and include at least 600 children of which parents will monitor disease incidence for six months. However, some DCCs and some parents/children might withdraw from the trial due to unforeseen reasons. To allow for about 15% lost to follow-up, we aim to include five extra intervention DCCs and five extra control DCCs (in total 35 intervention and 35 control DCCs).

### Process evaluation

A process evaluation will be conducted at the end of the trial (i.e. six months after start of the intervention) to identify strengths and weaknesses of the intervention, to be able to better interpret the results, and to provide recommendations for further intervention improvement. The process evaluation will include both qualitative and quantitative research. First, focus group discussions will be held with caregivers and managers of the intervention DCCs regarding their experience with the various components of the intervention. Second, a survey will be conducted to assess the extent to which caregivers and managers have been exposed to different intervention components, how workable and useful they found them, whether they liked them, and barriers or facilitators they experienced. Finally, another survey will be conducted to assess the effect of the intervention on sociocognitive determinants of caregivers’ HH compliance.

## Discussion

This paper outlines the study protocol for the evaluation of a DCC intervention aiming to increase caregivers’ and children’s HH compliance and decrease gastrointestinal and respiratory infections among children attending DCCs. Few DCC intervention studies have assessed, either caregivers’ or children’s HH compliance as outcome measure [13,15-17]. To our knowledge, this will be the first study to assess HH compliance of both caregivers and children as primary outcome measure and to report, besides overall compliance, the compliance for each specific HH indication. In addition, this will also be the first HH intervention in DCCs developed according to a stepwise behavioral approach [19] targeting the key determinants that underlie caregivers’ HH behavior. Although the intervention is based on determinants of HH compliance of caregivers, HH compliance of children will also be targeted and observed. Other strengths of the study are the randomized controlled design, the large number of participating DCCs and children/parents, and the long follow-up period. Furthermore, DCCs in the control group will also be offered the intervention after data collection, which probably will facilitate recruitment of participants and minimize dropout [13].

A possible limitation of the study is the Hawthorne effect when observing HH compliance, i.e. individuals might change their behavior when they know they are being observed [24,26]. However, during observations we will not inform caregivers that their HH is observed. If caregivers ask, they will be informed that the focus is on hygiene in general. Another possible limitation is that most caregivers will know, and parents and observers might recognize, the intervention status of the DCC. Data collection might be biased by this knowledge. In addition, illness will not be laboratory confirmed, which would be a more objective and specific outcome measure than monitoring of diarrhea and colds by parents. Furthermore, participating DCCs also participated in our previous study on determinants of caregivers’ HH compliance and will have received feedback on their HH compliance six months before start of the trial. Baseline compliance might therefore be higher than in DCCs who did not participate in our determinants study. The effect size that we will measure might then be an underestimation of the true effect size of the intervention.

This study will demonstrate whether our intervention is effective in improving compliance with HH guidelines and/or reducing gastrointestinal and respiratory infections among children in DCCs. The study can also provide insight into transmission of infectious diseases in DCCs (i.e. caregiver-to-child versus child-to-child transmission) and into changeable determinants of HH behavior of caregivers in DCCs. When an effect of the intervention is shown, (inter)national dissemination of the intervention in other DCCs may be considered. The intervention might then also be used by DCCs to distinguish them from a quality perspective and to control ongoing infectious disease outbreaks.

### Ethical approval

Ethical approval was waived by the Medical Ethics Committee of the Erasmus University Medical Center in Rotterdam (MEC-2011-256).

## Competing interests

The authors declare that they have no competing interests. Dispensers and refills will be sponsored by SCA Hygiene Products, Sweden.

## Authors’ contributions

All authors contributed to the design of the study and manuscript preparation. All authors have read and approved the final manuscript.

## Pre-publication history

The pre-publication history for this paper can be accessed here:

http://www.biomedcentral.com/1471-2334/13/259/prepub
